# Evaluation of perception towards brain health in Nigeria: Results from a nationwide awareness survey

**DOI:** 10.4102/jphia.v17i1.1270

**Published:** 2026-02-18

**Authors:** Temitope Farombi, Agustin Ibanez, Olajoke Akinyemi, Olufisayo Elugbadebo, Oluwagbemiga Oyinlola, Gabriel Ogunde, Joaquín Migeot, Chinedu Udeh-Momoh, Rufus Akinyemi

**Affiliations:** 1 Chief Tony Anenih Geriatric Center, University College Hospital, Ibadan, Nigeria; 2 Global Brain Health Institute (GBHI), Trinity College, Dublin, Ireland; 3 Brain Health Initiative Nigeria, Ibadan, Nigeria; 4 Department of Psychiatry, University College Hospital, Ibadan, Nigeria; 5 School of Social Work, McGill University, Montreal, Canada; 6 Department of Medical Social Services, University College Hospital, Ibadan, Nigeria; 7 Neuroscience and Ageing Research Unit, Institute of Advanced Medical Research and Training, University College Hospital, Ibadan, Nigeria; 8 Latin American Brain Health Institute (BrainLat), Universidad Adolfo Ibanez, Santiago, Nigeria; 9 School of Public Health Sciences, Wake Forest University School of Medicine, North Carolina, United States of America; 10 Brain and Mind Institute, Aga Khan University, Nairobi, Kenya; 11 Division of Clinical Geriatrics, Center for Alzheimer Research, Karolinska Institute, Stockholm, Sweden

**Keywords:** brain health, neurological disorders, Alzheimer’s disease, lifestyle factors, awareness, public perception, risk reduction, Nigeria

## Abstract

**Background:**

Brain health involves the continuous functioning of mental, cognitive, motor and physical abilities driven by brain processes. Despite high levels of brain health risk in Nigeria, there is a lack of data on the public perception of brain health.

**Aim:**

The authors investigated the perception of brain health and explored the interplay between demographic factors and brain health awareness.

**Setting:**

The research was carried out among the Nigerian population.

**Methods:**

A total of 570 participants responded to a cross-sectional survey conducted using Google Form link shared through WhatsApp and Facebook and convenience sampling between April 2023 and August 2023. Brain health perceptions were assessed across key domains. Statistical Package for Social Sciences version 29.0 was used for analysis. Bivariate correlations and logistic regression explored the relationships between socio-demographics and brain health perception.

**Results:**

Substance use was rated by 67% of participants as influencing factor for brain health. All life stages were considered important for brain care. Men were less likely than women to attribute family income, substance use and sleep as key influences. Remarkably, only 43.9%, 19.5% and 19.5% of participants agreed that an association exists between hypertension, diabetes and arthritis with brain health.

**Conclusion:**

The study’s findings suggest that there are notable gaps and gender differences in perceptions, underscoring the need for targeted health education. Addressing these gaps could improve the understanding of factors influencing brain health and support policy efforts in Nigeria.

**Contribution:**

This study provides unique insight into the gaps in the public perception of brain health in Nigeria, serving as a baseline study for future research.

## Introduction

Brain health, a fundamental component of overall well-being, reflects a complex, dynamic state of mental, motor, cognitive and functional abilities sustained by neurophysiological processes.^1^ Globally, brain disorders are now the leading cause of disability and the second leading cause of death, with disability-adjusted life years (DALY) attributable to neurological conditions rising by 18% from 1990 to 2021.^2^ This burden falls disproportionately on low- and middle-income countries, including Nigeria, where projections estimate a staggering 500% increase in neurological conditions by 2050, translating to 50.8 million DALYs and an economic impact of $51.4 billion.^3^

Demographic transitions, population growth, lifestyle behaviours, socio-economic factors and low education attainment are among the primary contributors to the increasing burden of brain disorders.^4^ A lack of awareness, limited knowledge and stigma associated with neurological conditions serve as significant barriers to diagnosis and treatment.^5,6^ In many low- and middle-income countries, particularly in sub-Saharan Africa, a common misconception is that brain disorders such as dementia are a natural part of ageing, associated with witchcraft and other negative spiritual connotations.^7,8^ However, accurate perceptions can promote healthier lifestyles,^9^ reducing the associated burden and stigma associated with these neurological conditions.

The significance of lifestyle factors demonstrated to impact brain health is poorly understood.^10^ Furthermore, little is known about public perceptions of brain health in Nigeria and the factors that influence it. Understanding these perceptions is essential for shaping public health strategies to mitigate modifiable risk factors, improve early detection and intervention, and guide educational and policy initiatives addressing Nigeria’s escalating neurological burden. It is vital to understand how people perceive brain health and the steps they are willing to take to preserve their brain functioning.^11^ Having this understanding would assist in determining the appropriate interventions to promote the right perceptions. Of note, the lack of policy, belief systems, and cultural values of individuals and communities contributes to the exacerbation of brain health issues.^12^ Given the high prevalence of brain disorders^13,14^ and lack of information on the Nigerian perception of brain health, it is imperative to investigate the public perception to identify knowledge gaps among our population.

The research question posed was: ‘How do socio-demographic factors shape public perception of brain health?’ This study aimed to capture a comprehensive view of perceptions regarding brain health in Nigeria. This study’s finding has the potential to inform the development of policies aimed at addressing brain health challenges in Nigeria. By understanding these influences, such policies could foster positive social and community impacts, ultimately enhancing overall well-being and contributing to societal progress.

## Research methods and design

### Study design and setting

This study employed a cross-sectional survey using social media platforms (WhatsApp and Facebook) to capture responses from the Nigerian population. This method was utilised because of its advantage of cost-effectiveness, having a wider reach, and rapidly covering diverse geographic areas within the shortest possible time.

### Sampling, study procedure and data collection

As this study was an exploratory baseline study, no prior sample size was calculated. The survey link was distributed via WhatsApp and Facebook to capture as many responses as possible within the period of April 2023 to August 2023 to provide a broad snapshot of public perceptions.

The target population included adults (aged ≥ 18 years) from different ethnic groups in Nigeria. At the beginning of the online questionnaire (Google Form), participants were asked to confirm their age (≥ 18 years) and indicate their ethnic group. Only individuals who met the age criterion and provided consent were able to proceed with completing the survey. To be eligible, participants had to be Nigerian residents, able to read in English, and able to use smartphones and other digital devices to complete the questionnaire. A non-random convenience sampling technique was used. A structured and previously validated questionnaire adopted from the Budin-Ljøsne et al.^11^ study was used to collect data from respondents. This self-administered questionnaire employed Likert and binary (yes/no) items to capture responses. A pretest of the study instrument was conducted using data from 50 respondents. Assessment of the instrument’s internal consistency yielded a Cronbach’s alpha coefficient of 0.776. The survey was available online from April 2023 to August 2023.

### Data collection

The questionnaire used for data collection comprised 11 questions on socio-demographic characteristics and three main questions that addressed the objective of the study. Socio-demographic assessment covered age, gender, highest educational qualification attained, marital status, ethnicity, profession, previous experience of participating in brain research, educational or work experience in healthcare, experience of long-standing illness, disability and health problem, experience of looking after a family member with brain disease, self-reported cognitive health (ability to think, remember and learn) and self-reported mental (ability to be mentally and emotionally balanced). The self-reported cognitive health and mental health were assessed based on participants’ perception of their status using the options provided (poor, below average, average, above average and excellent).

The first question probed the respondent’s perspective on the list of factors that impact brain health. This was performed using a 5-point Likert scale to rate each of the listed factors according to the extent to which they influence brain health. The Likert scale items were listed as very strong, strong, moderate, weak and no influence.

The second set of questions addressed respondents’ views on different life periods important for caring for the brain. A 4-point Likert scale (very important, important, moderately important, not important) was provided to rate how important each life period is.

The third question assessed respondents’ perception of a list of diseases and/or disorders associated with the brain. Respondents could either respond ‘yes’ or ‘no’ to express their view on which of the listed diseases/disorders is associated with the brain.

### Data analysis and management

The collected data were cleaned and analysed using Statistical Package for Social Sciences (SPSS) version 29.0. Descriptive statistics such as mean and standard deviation were used to summarise quantitative variables, while frequency and percentages were used for categorical variables. Inferential statistics using bivariate analysis were performed to investigate the association between socio-demographic variables and brain health awareness variables. Chi-square and Fisher’s exact test were performed for bivariate analysis where necessary. In addition, a logistic regression analysis was carried out to identify factors associated with brain health perception, dichotomised into strong influence (strong/very strong) and no influence (moderate/weak/no influence), important (very important/important) and not important (moderately important/not important), and yes and no as applicable to the perception questions. Odd ratios (OR) at 95% confidence intervals (CI) were reported for effect sizes. The analyses included only selected socio-demographic variables, specifically, age, gender, educational level, ethnicity, and work or educational experience in healthcare. The explanatory variables were socio-demographic characteristics measured through self-report in the online questionnaire. Age was categorised into three groups: 18–25 years, 26–40 years, 41–60 years, and ≥ 60 years. Gender was recorded as male or female. Educational level was categorised as special education, secondary education, vocational training and university and/or college degree. Ethnicity was classified into three main groups: Yoruba, Igbo and Hausa. Work or educational experience in healthcare was coded as a binary variable (yes or no). Study data were anonymised to protect participants’ personal information. Access to raw data was restricted to the principal investigator and designated analysts under strict confidentiality agreements. To ensure data accuracy and completeness, mandatory fields were included in the form to prevent missing responses.

### Ethical considerations

Ethical clearance to conduct this study was obtained from the Oyo State Ministry of Health Ethical Review Committee (No. AD 13/479/44509B). An informed consent form was provided at the beginning of the survey. Participants provided online written informed consent. All data were received through a password-protected gadget accessible to the researcher to maintain confidentiality.

## Results

Table 1 shows the socio-demographic characteristics of the 570 respondents who participated in the survey. Majority of the respondents were between the age range of 26–40 years (45.4%), female (61.4%), Yoruba (79.3%), had at least a University/college degree (89.9%), and single (72.1%).

**TABLE 1:** Socio-demographic characteristics of respondents.

Variable	Frequency	%
**Age (years)**		
18–25	148	26.0
26–40	259	45.4
41–60	103	18.1
> 60	60	10.5
**Gender**		
Male	220	38.6
Female	350	61.4
**Highest education attained qualification**		
Special education	6	1.1
Secondary education	42	7.4
Vocational training	10	1.8
University/College degree	512	89.8
**Marital status**		
Single	411	72.1
Married	150	26.3
Separated/divorced	1	0.2
Widowed	8	1.4
**Ethnicity**		
Yoruba	452	79.3
Igbo	92	16.1
Hausa	26	4.6
**Previous experience of participating in brain research**		
No	520	91.2
Yes	50	8.8
**Educational or work experience in healthcare**		
No	502	88.1
Yes	68	11.9
**Experience of long-standing illness, disability, or health problem**		
No	485	85.1
Yes	85	14.9
**Experience of looking after a family member with brain disease**		
No	502	88.1
Yes	68	11.9
**Self-reported cognitive health (ability to think, remember and learn)**		
Very poor	2	0.4
Below average	4	0.7
Average	48	8.4
Above average	201	35.3
Excellent	315	55.3
**Self-reported mental health (ability to be mentally and emotionally balanced)**		
Very poor	3	0.5
Below average	12	2.1
Average	73	12.8
Above average	203	35.6
Excellent	279	48.9

### Perception of factors influencing brain health

**FIGURE 1:**
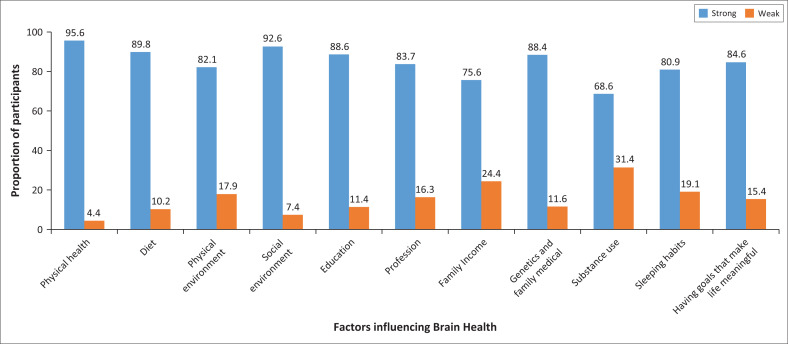
Factors influencing Brain Health. The figure shows the percentage of respondents that rank each factor ‘strong’ or ‘very strong’ (‘strong’ bar) and ‘moderate’ or ‘weak’ or ‘no influence’ (‘weak’ bar).

Figure 1 shows the proportion of respondents that rated each factor as having a strong or weak influence on brain health. Most respondents rated the following factors as having a strong influence on brain health; physical health (95.6%), social environment (92.6%), diet (89.8%), education (88.6%), genetics (88.4%), having life goals (84.6%), profession (83.7%), physical environment (82.1%) and sleeping habits (80.9%). Family income (75.6%) and substance abuse (68.6%) were rated by the respondents as a factor influencing brain health indicating a higher proportion who rated the two factors as a weak influence on brain health.

Table 2 shows that gender was significantly associated with perceptions of diet (χ^2^ = 4.69; *p* = 0.030), family income (χ^2^ = 4.30; *p* = 0.038), substance use (χ^2^ = 6.65; *p* = 0.010) and sleeping habits (χ^2^ = 5.71; *p* = 0.017) as an influencing factor for brain health.

**TABLE 2a:** Distribution pattern of public perception on factors influencing brain health.

Variable	Physical health							Diet						
	No influence		Strong influence		*p*	OR	95% CI	No influence		Strong influence		*p*	OR	95% CI
	*n*	%	*n*	%				*n*	%	*n*	%			
**Age (years)**														
18–25	6	4.1.	142	95.9	0.566	1.00	-	15	10.1	133	89.9	0.566	-	-
26–40	9	3.5	250	96.5	-	1.42	0.471–4.264	23	8.9	236	91.1	-	1.40	0.675–2.894
41–60	7	6.8	96	93.2	-	0.67	0.204–2.215	11	10.7	92	89.3	-	1.34	0.542–2.678
> 60	3	5.0	57	95.0	-	1.07	0.239–4.774	9	15.0	51	85.0	-	0.94	0.357–2.478
**Gender**														
Male	12	3.4	338	96.6	0.159	0.56	0.245–1.256	28	8.0	322	92.0	**0.030**	0.60	0.341–1.051
Female	13	5.9	207	94.1	-	-	-	30	13.6	190	86.4	-	-	-
**Highest educational level**														
Special training	0		6	100.0	0.742	-	-	2	33.3	4	66.7	0.170	-	-
Secondary education	1	2.4	41	97.6	-	0.00	-	3	7.1	39	92.7	-	8.66	0.916–81.871
Vocational training	0	-	10	100.0	-	0.83	-	2	20.0	8	80.0	-	2.42	0.151–38.763
University/college education	24	4.69	488	95.3	-	0.00	-	51	10.0	461	90.0	-	4.32	0.692–27.016
**Ethnicity**														
Hausa	3	11.1	24	88.9	0.203	-	-	9	33.3	18	66.7	< 0.001	-	-
Igbo	3	3.3	87	96.7	-	2.98	0.545–16.310	8	8.9	82	91.1	-	4.93	1.635–14.870
Yoruba	19	4.2	434	95.8	-	2.30	0.602–8.803	41	9.1	412	91.9	-	4.95	2.016–12.139
**WOE**														
No	-	-	-	-	-	-	-	-	-	-	-	-	-	-
Yes	-	-	-	-	-	0.44	0.191–0.993	-	-	-	-	-	0.67	0.377–1.183

Note: Bold values indicate statistically significant results (*p* < 0.05).

WOE, work or educational experience; CI, confidence intervals; OR, odds ratio.

**TABLE 2b:** Distribution pattern of public perception on factors influencing brain health.

Variable	Physical environment							Social environment						
	No influence		Strong influence		*p*	OR	95% CI	No influence		Strong influence		*p*	OR	95% CI
	*n*	%	*n*	%				*n*	%	*n*	%			
**Age (years)**														
18–25	28	18.9	120	81.1	0.659	1.00	-	14	9.5	134	90.5	0.670	1.00	-
26–40	41	15.8	218	84.2	-	1.32	0.757–2.312	16	6.2	243	93.8	-	1.52	0.680–3.389
41–60	20	19.4	83	80.6	-	1.05	0.529–2.071	8	7.8	95	92.2	-	1.38	0.507–3.767
> 60	13	21.7	47	78.3	-	0.98	0.451–2.143	4	6.7	56	93.3	-	1.75	0.507–6.042
**Gender**														
Female	58	16.6	292	83.4	0.299	-	-	23	6.6	327	93.4	0.358	-	-
Male	44	20.0	176	80.0	-	0.83	0.532–1.289	19	8.6	201	91.4	-	0.82	0.428–1.561
**Highest educational level**														
Special training	3	50.0	3	50.0	0.171	-	-	2	33.3	4	66.7	0.503	-	-
Secondary education	6	14.3	36	85.7	-	5.16	0.788–33.800	5	11.9	37	88.1		5.41	0.651–44.891
Vocational training	1	10.0	9	90.0	-	-	-	1	10.0	9	90.0	-	-	-
University/college education	92	18.0	420	82.0	-	4.07	0.771–21.536	34	6.6	478	93.4	-	7.56	1.200–47.684
**Ethnicity**														
Hausa	8	29.6	19	70.4	0.196	-	-	4	14.8	23	85.2	0.301	-	-
Igbo	13	14.4	77	85.6	-	2.46	0.882–6.843	7	7.8	83	92.2	-	2.23	0.586–8.438
Yoruba	81	17.9	373	82.1	-	1.88	0.784–4.513	31	6.8	422	93.4	-	2.57	0.813–8.114

CI, confidence intervals; OR, odds ratio.

**TABLE 2c:** Distribution pattern of public perception on factors influencing brain health.

Variable	Education							Profession						
	No influence		Strong influence		*p*	OR	95% CI	No influence		Strong influence		*p*	OR	95% CI
	*n*	%	*n*	%				*n*	%	*n*	%			
**Age (years)**														
18–25	19	12.8	129	87.2	0.095	1.00	-	20	13.5	128	86.5	0.189	-	-
26–40	23	8.9	236	91.1	-	1.34	0.670–2.662	38	14.7	221	85.3	-	0.81	0.431–1.506
41–60	11	10.7	92	89.3	-	1.11	0.480–2.563	21	20.4	82	79.6	-	0.56	0.273–1.150
> 60	12	20.0	48	80.0	-	0.51	0.220–1.188	14	23.3	46	76.7	-	0.46	0.206–1.021
**Gender**														
Female	40	11.4	310	88.6	0.981	-	-	55	15.7	295	84.3	0.624	-	-
Male	25	11.4	195	88.8	-	1.03	0.596–1.764	38	17.3	182	82.7	-	0.88	0.556–1.397
**Highest educational level**														
Special training	0	-	6	100.0	0.245	-	-	1	16.7	5	83.3	0.502	-	-
Secondary education	8	19.0	34	81.0	-	0.00	0	9	21.4	33	78.6	-	0.44	0.043–4.560
Vocational training	2	20.0	8	80.0	-	0.00	0	3	30.0	7	70.0	-	0.40	0.026–6.037
University/college education	55	10.7	437	89.3	-	0.00	0	80	15.6	432	84.4	-	0.75	0.083–6.707
**Ethnicity**														
Hausa	5	18.5	22	81.5	0.266	-	-	6	22.2	21	77.8	0.616	-	-
Igbo	13	14.4	77	85.6	-	1.38	0.434–4.388	16	17.8	74	82.2	-	1.26	0.432–3.659
Yoruba	47	10.4	406	89.6	-	1.90	0.671–5.403	-	-	382	84.3	-	1.36	0.524–3.553

CI, confidence intervals; OR, odds ratio.

**TABLE 2d:** Distribution pattern of public perception on factors influencing brain health.

Variable	Family income							Genetics and family medical history						
	No influence		Strong influence		*p*	OR	95% CI	No influence		Strong influence		*p*	OR	95% CI
	*n*	%	*n*	%				*n*	%	*n*	%			
**Age (years)**														
18–25	39	26.3	109	73.7	0.748	-	-	17	11.5	131	88.5	0.507	-	-
26–40	61	23.5	198	76.5	-	1.12	0.683–1.834	30	11.6	229	88.4	-	0.93	0.475–1.831
41–60	27	26.2	76	73.8	-	0.89	0.487–1.634	9	8.7	94	91.3	-	1.14	0.471–2.776
> 60	12	20.0	48	80.0	-	1.35	0.631–2.880	10	16.7	50	83.3	-	0.59	0.241–1.421
**Gender**														
Female	75	21.4	275	78.6	0.038	-	-	37	10.6	313	89.4	0.343	-	-
Male	64	29.1	156	70.9	-	0.67	0.450–0.984	29	13.2	191	86.8	-	0.76	0.451–1.288
**Highest educational level**														
Special training	1	16.7	5	83.3	0.930	-	-	0	-	6	100.0	0.613†	-	-
Secondary education	11	26.2	31	73.8	-	0.47	0.047–4.724	6	14.3	36	85.7	-	0.00	0
Vocational training	3	30.0	7	70.0	-	1.22	0.059–25.167	2	20.0	8	80.0	-	0.74	0
University/college education	124	24.2	388	75.8	-	0.55	0.061–4.860	58	11.3	454	88.7	-	0.00	0
**Ethnicity**														
Hausa	9	33.3	18	66.7	0.428	-	-	3	11.1	24	88.9	0.871	-	-
Igbo	19	21.1	71	78.9	-	1.81	0.696–4.715	9	10.0	81	90.0	-	1.01	0.249–4.081
Yoruba	111	24.5	342	75.5	-	1.49	0.644–3.466	54	11.9	399	88.1	-	0.80	0.229–2.815

CI, confidence intervals; OR, odds ratio.

**TABLE 2e:** Distribution pattern of public perception on factors influencing brain health.

Variable	Substance use							Sleeping habit						
	No influence		Strong influence		*p*	OR	95% CI	No influence		Strong influence		*p*	OR	95% CI
	*n*	%	*n*	%				*n*	%	*n*	%			
**Age (years)**														
18–25	51	34.5	97	65.5	0.19	-	-	33	22.3	115	77.7	0.189	-	-
26–40	87	33.6	172	66.4	-	1.06	0.673–1.665	41	15.8	218	84.2	-	1.56	0.910–2.687
41–60	28	27.2	75	72.8	-	1.55	0.855–2.809	25	24.3	78	75.7	-	0.91	0.481–1.719
> 60	13	21.7	47	78.3	-	1.92	0.926–3.971	10	16.7	50	83.3	-	1.58	0.695–3.570
**Gender**														
Female	96	27.4	254	72.6	**0.010**	-	-	56	16.0	294	84.0	**0.017**	-	-
Male	83	37.7	137	62.3	-	0.63	0.435–0.904	53	24.1	167	75.9	-	0.61	0.394–0.927
**Highest education level**														
Special training	2	33.3	4	66.7	0.067	-	-	2	33.3	4	66.7	0.806	-	-
Secondary education	12	28.6	30	71.4	-	1.81	0.273–11.952	9	21.4	33	78.6	-	1.89	0.272–13.165
Vocational training	7	70.0	3	30.0	-	0.21	0.019–.282	2	20.0	8	80.0	-	2.92	0.188–45.562
University/college education	158	30.9	354	69.1	-	1.36	0.236–7.788	96	18.7	416	81.3	-	1.66	0.283–9.712
**Ethnicity**														
Hausa	7	25.9	20	74.1	0.676	-	-	7	25.9	20	74.1	0.654	-	-
Igbo	26	28.9	64	71.1	-	0.86	0.320–2.319	17	18.9	73	81.1	-	1.43	0.512–3.992
Yoruba	146	32.2	307	67.8	-	0.78	0.318–1.927	85	18.8	368	81.2	-	1.43	0.575–3.574

Note: Bold values indicate statistically significant results (*p* < 0.05).

CI, confidence intervals; OR, odds ratio.

**TABLE 2f:** Distribution of perception of specific periods to look after one’s brain.

Variable	Before Birth							Childhood						
	Not important		Important		*p*	OR	95% CI	Not important		Important		*p*	OR	95% CI
	*n*	%	*n*	%				*n*	%	*n*	%			
**Age category (years)**														
18–25	29	19.6	119	80.4	0.421	-	-	3	2.0	145	98.0	0.423	-	-
26–40	-	-	-	-	-	0.84	0.49–1.46	9	3.5	250	96.5	-	0.38	0.09–1.65
41–60	-	-	-	-	-	1.30	0.62–2.75	6	5.8	97	94.2	-	0.24	0.05–1.11
> 60	-	-	-	-	-	0.67	0.31–1.44	3	5.0	57	95.0	-	0.28	0.05–1.62
**Gender**														
Female	54	15.4	296	84.6	**0.002**	-	-	13	3.7	337	96.3	0.962	-	-
Male	57	25.9	163	74.1	-	0.54	0.35–0.83	8	3.6	212	96.4	-	1.01	0.40–2.23
**Highest educational level**														
Special training	4	66.7	2	33.3	**0.006**†	-	-	1	16.7	5	83.3	0.184†	-	-
Secondary education	11	26.2	31	73.8	-	5.66	0.80–40.23	3	7.1	39	92.9		0.10	0.07–13.68
Vocational training	0		10	100.0	-	-	-	0	-	10	100.0	-	-	-
University/college education	96	18.8	416	81.2	-	9.04	1.48–55.28	17	3.7	495	96.7	-	3.81	0.38–37.86
**Ethnicity**														
Hausa	5	18.5	22	81.5	0.409	-	-	1	3.7	26	96.3	0.982	-	-
Igbo	13	14.4	77	85.6	-	1.27	0.40–4.03	3	3.3	87	96.7	-	1.18	0.02–12.15
Yoruba	93	20.5	360	79.5	-	0.81	0.29–2.24	17	3.7	436	96.3	-	0.89	0.11–7.11

Note: Bold values indicate statistically significant results (*p* < 0.05).

CI, confidence intervals; OR, odds ratio.

**TABLE 2g:** Distribution of perception of specific periods to look after one’s brain.

Variable	Adolescence							Young adulthood						
	No influence		Strong influence		*p*	OR	95% CI	No influence		Strong influence		*p*	OR	95% CI
	*n*	%	*n*	%				*n*	%	*n*	%			
**Age (years)**														
18–25	2	1.3	146	98.7	**0.003**	-	-	5		143	96.6	**0.036**	-	-
26–40	3	1.2	256	98.8	-	1.19	0.16–8.11	9	3.5	250	96.5	-	0.77	0.23–2.61
41–60	8	7.8	95	92.2	-	0.22	0.04–1.25	4	3.9	99	96.1	-	0.84	0.19–3.73
> 60	2	3.3	58	96.7	-	0.58	0.07–4.90	7	11.7	53	88.3	-	0.25	0.07–0.93
**Gender**														
Female	10	2.9	340	97.1	0.671	-	-	13	3.7	337	96.3	0.323	-	-
Male	5	2.3	215	97.7	-	1.51	0.46–4.88	12	5.4	208	94.6	-	0.76	0.33–1.75
**Highest educational level**														
Special training	2	33.3	4	66.7	**< 0.001**†	-	-	2	33.3	4	66.7	**0.004**†	-	-
Secondary education	1	2.4	41	97.6	-	11.21	0.59–213.56	3	7.1	39	92.9	-	3.94	0.37–41.52
Vocational training	0		10	100.0	-	-	-	0	-	10	100.0	-		
University/college education	12	2.3	500	97.7	-	13.55	1.87–98.05	20	3.9	492	96.1	-	9.32	1.30–66.67
**Ethnicity**														
Hausa	2	7.4	25	95.6	0.235	-	-	2	7.4	25	92.6	0.730	-	-
Igbo	3	3.3	87	96.7	-	2.38	0.35–16.35	4	4.4	86	95.6	-	1.67	0.28–10.07
Yoruba	10	2.2	443	97.8	-	3.13	0.59–16.50	19	4.2	434	95.8	-	1.63	0.34–7.76

Note: Bold values indicate statistically significant results (*p* < 0.05).

CI, confidence intervals; OR, odds ratio.

**TABLE 2h:** Distribution of perception of specific periods to look after one’s brain.

Variable	Middle age							Old age						
	No influence		Strong influence		*p*	OR	95% CI	No influence		Strong influence		*p*	OR	95% CI
	*n*	%	*n*	%				*n*	%	*n*	%			
**Age (years)**														
18–25	15	10.1	133	89.9	0.229	-	-	22	14.9	126	85.1	0.615	-	-
26–40	22	8.5	237	91.5	-	1.27	0.62–2.62	45	17.4	214	82.6	-	0.85	0.47–1.52
41–60	16	15.5	87	84.5	-	0.63	0.28–1.41	22	21.4	81	78.6	-	0.64	0.32–1.28
> 60	8	13.3	52	86.7	-	0.81	0.31–2.11	11	18.3	49	81.7	-	0.80	0.35–1.84
**Gender**														
Female	35	10.0	315	90.0	0.494	-	-	55	15.7	295	84.3	0.147	-	-
Male	26	11.8	194	88.2	-	0.84	0.49–1.46	45	20.4	175	79.6	-	0.72	0.46–1.12
**Highest educational level**														
Special training	2	33.3	4	66.7	0.348	-	-	2	33.3	4	66.7	0.625	-	-
Secondary training	4	9.5	38	90.5	-	3.36	0.42–26.95	6	14.3	36	85.7	-	2.13	0.30–15.37
Vocational training	1	10.0	9	90.0	-	-	-	1	10.0	9	90.0	-	2.75	0.18–42.09
University/college education	54	10.5	458	89.5	-	2.88	0.49–16.92	91	17.8	421	82.2	-	1.80	0.31–10.38
**Ethnicity**														
Hausa	5	18.5	22	81.5	-	-	-	6	22.2	21	77.8	0.594	-	-
Igbo	8	8.9	82	91.1	-	2.29	0.67–7.83	13	14.4	77	85.6	-	1.58	0.53–4.70
Yoruba	48	10.6	405	89.4	-	1.79	0.63–5.04	81	17.9	372	82.1	-	1.20	0.46–3.11

CI, confidence intervals; OR, odds ratio.

Logistic regression analysis (Table 2) shows that men had 33% lower odds to consider family income (OR = 0.67, 95% CI: 0.45–0.98), 37% lower odds to consider substance use (OR = 0.63, 95% CI: 0.44–0.98), and 39% lower odds to consider sleeping habits (OR = 0.61, 95% CI: 0.39–0.93) as important factors influencing brain health compared with women. Ethnicity was also significantly associated with perceptions of diet (χ^2^ = 16.63; *p* < 0.001). Specifically, the odds of Yoruba respondents to consider diet as an important factor were almost five times higher (OR = 4.95, 95% CI: 2.02–12.14), and the odds among Igbo respondents were similarly about five times higher (OR = 4.93, 95% CI: 1.64–14.87), compared with Hausa respondents. In addition, respondents with university education had more than seven times the odds of considering social environment as an important factor for brain health compared with those with lower educational levels (OR = 7.56, 95% CI: 1.20–47.68) (Table 2).

### Public perception of specific life periods to look after one’s brain

**FIGURE 2:**
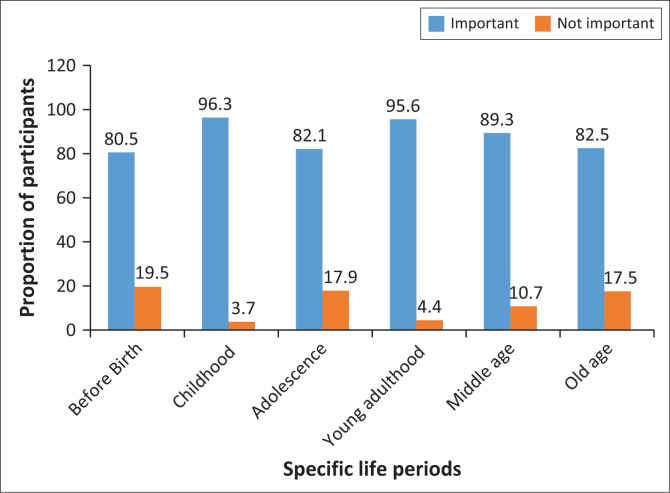
Specific life periods to look after one’s brain. The figure shows the percentage of respondents rating each specific life period as ‘very important’ or ‘important’ (‘important’ bar) and ‘moderately important’ or ‘not important’ (‘not important’ bar).

The results in Figure 2 show that all life periods were rated as important to look after, before birth (80.5%), childhood (96.3%), adolescence (82.1%), young adulthood (95.6%), middle age (89.3%) and old age (82.5%).

Bivariate analysis variables of gender (χ^2^ = 9.6; *p* = 0.002) and highest educational level (χ^2^ = 12.31; *p* = 0.006) were significantly associated with the belief that the period ‘before birth’ is important for looking after one’s brain. Men were less likely than women to rate before birth (OR = 0.54, 95% CI: 0.35–0.83) as a life period important for taking care of the brain. Age is significantly associated with the perception that adolescence (χ^2^ = 13.85; *p* = 0.003) and young adulthood (χ^2^ =8.51; *p* = 0.036) are periods to take care of one’s brain. Similarly, educational level is associated with the perception of adolescence (χ^2^ = 22.51; *p* < 0.001) and young adulthood (χ^2^ = 13.48; *p* = 0.004) as important life periods for brain health care (Table 2).

Respondents aged over 60 years had 75% lower odds of considering young adulthood as an important life period for looking after one’s brain compared with younger age groups (OR = 0.25, 95% CI: 0.07–0.93). They were also less likely overall to regard all life periods as important for brain health. In contrast, respondents with university education had significantly higher odds of recognising key developmental stages as critical for brain health. Specifically, they had 9 times higher odds of rating the period before birth (OR = 9.04, 95% CI: 1.48–55.28), over 13 times higher odds of rating adolescence (OR = 13.55, 95% CI: 1.87–98.05), and approximately nine times higher odds of rating young adulthood (OR = 9.32, 95% CI: 1.30–66.67) as important for taking care of the brain compared with respondents of lower educational levels (Table 2).

### Public perception of diseases and disorders associated with the brain

**FIGURE 3:**
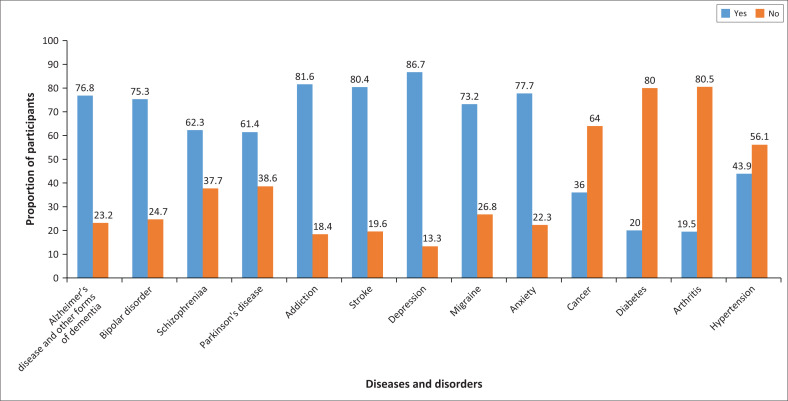
Showing percentage of respondents’ responses on diseases/disorders associated with the brain.

Figure 3 shows the proportion of respondents based on their perception of diseases and disorders associated with brain health. The majority of the respondents perceived that the following diseases/disorders are associated with brain health: addiction (81.6%), stroke (80.4%) and depression (86.7%). Most respondents perceived that Alzheimer’s disease and other forms of dementia (76.8%), bipolar disorder (75.3%), migraine (73.2%), anxiety (77.7%), schizophrenia (62.3%) and Parkinson’s disease (61.4%) are associated with brain health. Compared to the above-mentioned responses, a lower percentage of respondents perceived that cancer (36%), diabetes (19.5%), arthritis (19.5%) and hypertension (43.9%) are associated with the brain.

Table 3 revealed that age was found to be significantly associated with perception on bipolar disorder (χ^2^ = 17.4; *p* = 0.001), schizophrenia (χ^2^ = 9.37; *p* = 0.025), Parkinson disease (χ^2^ = 12.65; *p* = 0.005), addiction (χ^2^ = 22.56; *p* < 0.001), stroke (χ^2^ = 14.85; *p* = 0.002), migraine (χ^2^ = 20.59; *p* < 0.001), anxiety (χ^2^ = 14.65; *p* = 0.002) and hypertension (χ^2^ = 10.92; *p* = 0.012).

**TABLE 3a:** Distribution pattern of public perception on diseases and disorders associated with the brain.

Variable	AD and other forms of dementia							Schizophrenia						
	No		Yes		*p*	OR	95% CI	No		Yes		*p*	OR	95% CI
	*n*	%	*n*	%				*n*	%	*n*	%			
**Age category (years)**														
18–25	36	24.3	112	75.7	0.100	-	-	57	38.5	91	61.5	**0.025**	-	-
26–40	52	20.1	207	79.9	-	1.34	0.80–2.24	91	35.1	168	64.9	-	1.25	0.78–1.96
41–60	23	22.3	80	77.7	-	1.22	0.64–2.34	34	33.0	69	67.0	-	1.56	0.87–2.78
> 60	21	35.0	39	65.0	-	0.58	0.29–1.16	33	55.0	27	45.0	-	0.52	0.28–0.10
**Gender**														
Female	64	18.3	286	81.7	**0.001**	-	-	119	34.0	231	66.0	**0.021**	-	-
Male	68	30.9	152	69.1	-	0.50	0.33–0.74	96	43.6	124	56.4	-	0.68	-
Highest educational level														
Special training	3	50.0	3	50.0	0.058	-	-	4	66.7	2	33.3	**0.044**	-	-
Secondary education	7	16.7	35	83.3	-	4.06	0.60–27.46	12	28.6	30	71.4	-	5.18	0.75–35.63
Vocational training	5	50.0	5	50.0	-	0.51	0.05–4.96	7	70.0	3	30.0	-	0.24	0.02–3.80
University/college education	117	22.8	395	77.2	-	2.50	0.45–13.80	192	37.5	320	62.5	-	3.23	0.54–19.40
**Ethnicity**														
Hausa	7	25.9	20	74.1	0.885	-	-	15	55.6	12	44.4	0.141	-	-
Igbo	22	24.4	68	75.6	-	1.03	0.37–2.86	32	35.6	58	64.4	-	2.40	0.97–5.95
Yoruba	103	22.7	350	77.3	-	1.16	0.46–2.92	168	37.1	285	62.9	-	2.28	1.00–5.16

Note: Bold values indicate statistically significant results (*p* < 0.05).

AD, alzheimer’s disease; CI, confidence intervals; OR, odds ratio.

**TABLE 3b:** Distribution pattern of public perception on diseases and disorders associated with the brain.

Variable	Bipolar disease							Parkinson’s disease						
	No		Yes		*p*	OR	95% CI	No		Yes		*p*	OR	95% CI
	*n*	%	*n*	%				*n*	%	*n*	%			
**Age category (years)**														
18–25	34	23.0	114	77.0	**0.001**	-	-	57	38.5	91	61.5	**0.005**	-	-
26–40	56	21.6	203	78.4	-	1.10	0.65–1.83	85	32.8	174	67.2	-	0.79	0.43–1.45
41–60	23	22.3	80	77.7	-	1.10	0.57–2.12	44	42.7	59	57.3	-	0.64	0.31–1.33
> 60	28	46.7	32	53.3	-	0.30	0.15–0.60	34	56.7	26	43.3	-	0.23	0.11–0.49
**Gender**														
Female	76	21.7	274	78.3	**0.035**	-	-	124	35.4	226	64.6	**0.050**	-	-
Male	65	30.0	155	70.0	-	0.66	0.44–0.99	96	43.6	124	56.4	-	0.71	0.45–1.10
**Highest educational level**														
Special training	3	5.0	3	50.0	0.100	-	-	5	83.3	1	16.7	**0.016**	-	-
Secondary education	8	19.1	34	80.9	-	2.91	0.43–19.87	13	30.9	29	69.1	-	3.90	0.54–28.11
Vocational training	5	50.0	5	50.0	-	0.46	0.05–4.72	7	70.0	3	30.0	-	3.62	0.24–53.91
University/college education	125	24.4	387	75.6	-	2.34	0.41–13.34	195	38.1	3	30.0	-	2.71	0.48–15.02
**Ethnicity**														
Hausa	9	33.3	18	66.7	0.502	-	-	11	40.7	16	59.3	0.832	-	-
Igbo	20	22.2	70	77.8	-	1.76	0.65–4.74	37	41.1	53	58.9	-	0.67	0.22–2.07
Yoruba	112	24.7	341	75.3	-	1.48	0.62–3.57	172	38.0	281	62.0	-	0.85	0.30–2.41

Note: Bold values indicate statistically significant results (*p* < 0.05).

CI, confidence intervals; OR, odds ratio.

**TABLE 3c:** Distribution pattern of public perception on diseases and disorders associated with the brain.

Variable	Addiction							Stroke						
	No		Yes		*p*	OR	95% CI	No		Yes		*p*	OR	95% CI
	*n*	%	*n*	%				*n*	%	*n*	%			
**Age category (years)**														
18–25	19	12.8	129	87.2	**< 0.001**	-	-	21	14.2	127	85.8	**0.002**	-	-
26–40	42	16.2	217	83.8	-	1.34	0.670–2.662	46	17.8	213	82.2	-	0.73	0.41–1.32
41–60	20	19.4	83	806	-	1.11	0.480–2.563	23	22.3	80	77.7	-	0.58	0.29–1.19
> 60	24	40.0	36	60.0	-	0.51	0.220–1.188	22	36.7	38	63.3	-	0.27	0.13–0.57
**Gender**														
Female	58	16.6	292	83.4	0.151	-	-	61	17.4	289	82.6	0.092	-	-
Male	47	21.4	173	78.6	-	1.03	0.596–1.764	51	23.2	169	76.8	-	0.69	0.45–1.06
**Highest educational level**														
Special training	3	50.0	3	50.0	0.128	-	-	4	66.7	2	33.3	**0.010**	-	-
Secondary education	5	11.9	37	88.1	-	0.00	0	7	16.7	35	83.3	-	5.16	0.72–37.24
Vocational training	1	10.0	9	90.0	-	0.00	0	4	40.0	6	60.0	-	3.36	0.31–36.83
University/college education	96	18.7	416	81.3	-	0.00	0	97	18.9	415	81.1	-	5.40	0.91–32.04
**Ethnicity**														
Hausa	5	18.5	22	81.5	0.594	-	-	5	18.5	22	81.5	0.986	-	-
Igbo	20	22.2	70	77.8	-	1.38	0.434–4.388	18	20.0	72	80.0	-	0.81	0.26–2.52
Yoruba	80	17.7	373	82.3	-	1.90	0.671–5.403	89	19.6	364	80.4	-	0.78	0.28–2.19

Note: Bold values indicate statistically significant results (*p* < 0.05).

CI, confidence intervals; OR, odds ratio.

**TABLE 3d:** Distribution pattern of public perception on diseases and disorders associated with the brain.

Variable	Depression							Migraine						
	No		Yes		*p*	OR	95% CI	No		Yes		*p*	OR	95% CI
	*n*	%	*n*	%				*n*	%	*n*	%			
**Age category (years)**														
18–25	21	14.2	127	85.8	0.080	-	-	32	21.6	116	78.4	**< 0.001**	-	-
26–40	28	10.8	231	89.2	-	1.51	0.81–2.83	57	22.0	202	78.0	-	0.93	0.55–1.57
41–60	13	12.6	90	87.4	-	1.28	0.89–2.80	36	35.0	67	65.0	-	0.54	0.29–0.99
> 60	14	23.3	46	76.7	-	0.59	0.27–1.30	28	46.7	32	53.3	-	0.30	0.15–0.60
**Gender**														
Female	44	12.6	306	87.4	0.500	-	-	82	23.4	268	76.6	**0.020**	-	-
Male	32	14.5	188	85.5	-	0.85	0.52–1.40	71	32.3	149	67.7	-	0.64	0.43–0.94
**Highest educational level**														
Special training	2	33.3	4	66.7	0.277	-	-	5	83.3	1	16.7	0.013	-	-
Secondary education	3	7.1	39	92.9	-	5.78	0.66–50.47	11	16.2	31	73.8	-	6.99	0.69–71.06
Vocational training	2	20.0	8	80.0	-	2.28	0.14–35.686	4	40.0	6	60.0	-	2.51	0.18–35.10
University/college education	69	13.5	443	86.5	-	2.49	0.41–14.10	133	26.0	379	74.0	-	8.25	0.92–74.30
**Ethnicity**														
Hausa	3	11.1	24	88.9	0.941	-	-	11	40.7	16	59.3	0.246	-	-
Igbo	12	13.3	78	86.7	-	0.80	0.20–3.13	24	26.7	66	73.3	-	1.80	0.71–4.57
Yoruba	61	13.5	392	86.5	-	0.78	0.22–2.75	118	26.0	335	74.0	-	1.73	0.76–3.96

Note: Bold values indicate statistically significant results (*p* < 0.05).

CI, confidence intervals; OR, odds ratio.

**TABLE 3e:** Distribution pattern of public perception on diseases and disorders associated with the brain.

Variable	Anxiety							Cancer						
	No		Yes		*p*	OR	95% CI	No		Yes		*p*	OR	95% CI
	*n*	%	*n*	%				*n*	%	*n*	%			
**Age category (years)**														
18–25	29	19.6	119	80.4	0.002	-	-	89	60.1	59	39.9	0.107	-	-
26–40	46	17.8	213	82.2	-	1.05	0.61–1.81	165	63.7	94	36.3	-	0.91	0.59–1.41
41–60	29	28.2	74	71.8	-	0.58	0.31–1.09	76	73.8	27	26.2	-	0.57	0.32–1.02
> 60	23	38.3	37	61.7	-	0.35	0.18–0.71	35	58.3	25	41.7	-	-	-
**Gender**														
Female	77	22.0	273	78.0	0.839	-	-	229	65.4	121	34.6	0.382	-	-
Male	50	22.7	170	77.3	-	0.94	0.62–1.43	136	61.8	84	38.2	-	1.13	0.79–1.61
**Highest educational level**														
Special training	4	66.7	2	33.3	0.071	-	-	4	6.7	2	33.3	0.610	-	-
Secondary education	10	23.8	32	76.2	-	3.36	0.50–22.722	23	54.8	19	45.2	-	1.24	0.19–8.16
Vocational training	2	20.0	8	80.0	-	9.05	0.59–139.87	7	70.0	3	30.0	-	0.90	0.08–9.10
University/college education	111	21.7	401	78.3	-	4.30	0.74–24.73	331	64.6	181	35.4	-	0.94	0.16–5.51
**Ethnicity**														
Hausa	4	14.8	23	85.2	0.625	-	-	17	63.0	10	37.0	0.722	-	-
Igbo	21	23.3	69	76.7	-	0.55	0.17–1.83	61	67.8	29	32.2	-	0.81	0.33–2.02
Yoruba	102	22.5	351	77.5	-	0.53	0.18–1.62	287	63.4	166	36.6	-	0.98	0.43–2.2

CI, confidence intervals; OR, odds ratio.

**TABLE 3f:** Distribution pattern of public perception on diseases and disorders associated with the brain.

Variable	Diabetes							Arthritis						
	No		Yes		*p*	OR	95% CI	No		Yes		*p*	OR	95% CI
	*n*	%	*n*	%				*n*	%	*n*	%			
**Age category (years)**														
18–25	122	82.4	26	17.6	0.125	-	-	126	85.1	22	14.9	0.401	-	-
26–40	213	82.2	46	17.8	-	1.04	0.59–1.85	205	79.1	54	20.9	-	1.65	0.92–2.99
41–60	74	71.8	29	28.2	-	1.72	0.90–3.32	82	79.6	21	20.4	-	1.34	0.65–2.74
> 60	47	78.3	13	21.7	-	1.21	0.55–2.67	46	76.7	14	23.3	-	1.75	0.79–3.87
**Gender**														
Female	270	77.1	80	22.9	**0.031**	-	-	273	78.0	77	22.0	0.055	-	-
Male	186	84.5	34	15.5	-	0.59	0.28–0.93	186	84.5	34	15.5	-	0.62	0.39–0.97
**Highest educational level**														
Special training	3	50.0	3	50.0	0.273	-	-	4	66.7	2	33.3	0.549		
Secondary education	32	76.2	10	23.8	-	0.35	0.05–2.23	32	76.2	10	23.8	-	0.65	0.10–4.46
Vocational training	8	80.0	2	20.0	-	0.35	0.03–3.70	7	70.0	3	30.0	-	1.51	0.15–14.95
University/college education	413	80.7	99	19.3	-	0.25	0.05–1.37	416	81.2	96	18.8	-	0.38	0.07–2.23
**Ethnicity**														
Hausa	21	77.8	6	22.2	0.674	-	-	20	74.1	7	25.9	0.685	-	-
Igbo	75	83.3	15	16.7	-	0.66	0.22–1.96	73	81.1	17	18.9	-	0.63	0.23–1.74
Yoruba	360	79.5	93	20.5	-	0.94	0.36–2.46	366	80.8	87	19.2	-	0.66	0.27–1.65

Note: Bold values indicate statistically significant results (*p* < 0.05).

CI, confidence intervals; OR, odds ratio.

**TABLE 3g:** Distribution pattern of public perception on diseases and disorders associated with the brain.

Variable	Hypertension						
	No		Yes		*p*	OR	95% CI
	*n*	%	*n*	%			
**Age category (years)**							
18–25	97	65.5	51	34.5	**0.012**	-	-
26–40	143	55.2	116	44.8	-	1.44	0.92–2.23
41–60	46	44.7	57	55.3	-	2.06	1.20–3.54
> 60	34	56.7	26	43.3	-	1.29	0.69–2.44
**Gender**							
Female	188	53.7	162	46.3	0.141	-	-
Male	132	60.0	88	40.0	-	0.79	0.56–1.12
**Highest educational level**							
Special training	2	33.3	4	66.7	0.228	-	-
Secondary education	29	69.1	13	30.9	-	0.34	0.05–2.22
Vocational training	6	60.0	4	40.0	-	0.63	0.06–6.32
University/college education	283	55.3	229	44.7	-	0.46	0.08–2.64
**Ethnicity**							
Hausa	14	51.8	13	48.2	0.831	-	-
Igbo	49	54.4	41	45.6	-	0.91	0.38–2.19
Yoruba	257	56.7	196	43.3	-	0.86	0.39–1.90

Note: Bold values indicate statistically significant results (*p* < 0.05).

CI, confidence intervals; OR, odds ratio.

Compared with younger age groups, respondents aged over 60 years had significantly lower odds of viewing several conditions as brain-related: bipolar disorder (70% lower odds; OR = 0.30, 95% CI: 0.15–0.60), Parkinson’s disease (77% lower odds; OR = 0.23, 95% CI: 0.11–0.49), addiction (73% lower odds; OR = 0.27, 95% CI: 0.13–0.57), migraine (70% lower odds; OR = 0.30, 95% CI: 0.15–0.60) and anxiety (82% lower odds; OR = 0.18, 95% CI: 0.05–0.71) (Table 3). Respondents aged 41–60 years, however, had higher odds of rating hypertension as a disease associated with brain health. Educational level was significantly associated with the perception of schizophrenia (χ^2^ = 8.08, *p* = 0.044), Parkinson’s disease (χ^2^ = 10.31, *p* = 0.016), stroke (χ^2^ = 11.42, *p* = 0.010) and migraine (χ^2^ = 10.83, *p* = 0.013). Gender differences were also observed, with men having significantly lower odds than women of perceiving Alzheimer’s disease (OR = 0.50, 95% CI: 0.33–0.74), bipolar disorder (OR = 0.66, 95% CI: 0.44–0.99), schizophrenia (OR = 0.68, 95% CI: 0.46–0.98), migraine (OR = 0.64, 95% CI: 0.43–0.94), diabetes (OR = 0.59, 95% CI: 0.28–0.93) and arthritis (OR = 0.62, 95% CI: 0.39–0.97) as diseases associated with the brain (Table 3).

## Discussion

This study provides unique insight into the public perception of brain health in Nigeria, serving as a baseline study for future research. In descending order, respondents rated the importance of factors influencing brain health as follows: physical health, social environment, diet, education, genetics, life goals, profession, physical environment, sleeping habits, family income and substance use. Surprisingly, quite a significant proportion of our respondents rated substance use as a weak factor influencing brain health. Men were less likely to view substance use as a factor that has an impact on brain health. We observed that all life periods, with more emphasis on childhood and young adulthood, were rated as important for taking care of the brain. The diseases and disorders highly rated as having an association with brain health include migraine, bipolar disorder, Alzheimer’s disease, anxiety, stroke, addiction and depression in an ascending order. Remarkably, a large percentage disagreed on the association of hypertension, diabetes and arthritis with brain health.

Substance use disorders are identified as important global contributors to disability and mortality.^15^ A sizable portion of our respondents did not perceive that substance use could impact brain health. This result indicated a contrast with studies conducted in Western countries, which reported a high ranking of substance use as an influencing factor for brain health.^11,16^ Substance use at high doses leads to consequences such as embarrassment, automobilist accidents, sexual abuse, child abuse, suicide attempts and fatalities, cardiovascular accidents, or overdose death.^17^ In developing countries such as Nigeria, the necessity of raising awareness and sensitisation on substance use, which has been demonstrated to hurt brain health,^18^ cannot be over-emphasised.

The majority of our respondents believed that sleep is an important factor for brain health. This finding corroborates with previous research that identified poor sleep quality impacts different domains including physical, psychological and cognitive health.^19,20^ Similar to the findings from Watson et al.’s^21^ study, our study showed a significant association between gender and perception of sleep.^21^ Specifically, men were less likely to perceive that sleep is associated with brain health. This may be because of societal norms that make men place more importance on work than on getting enough sleep. For instance, gender inequity in Nigeria consequently allows more men than women to participate in jobs such as jobs in the manufacturing sector.^22^ Combating gender inequity and creating targeted awareness could indirectly address this uninformed perception.

Our findings indicated that ethnicity significantly influenced the perception of our respondents on the link between diet and brain health. Disparities in diverse cultures and dietary preferences may influence the perception of different ethnic groups. Further analysis showed that older adults were less likely than younger ones to perceive diet as an influencing factor for brain health. Similarly, older adults of different ethnic groups in other parts of the world, including Hispanics, whites and African Americans, have previously expressed doubt on the relationship between brain health and diet.^23^ Our respondents rated physical health as the highest influencing factor of brain health. This high rating is consistent with a report from Budin-Ljøsne and colleagues,^11^ which implies that physical health seems to be held with high importance across diverse geographical contexts.

We observed that men were less likely to perceive the ‘before birth’ period as a phase that needs proper care for the brain. This could be linked to less male involvement and awareness of preconception and conception care practices in developing nations.^24,25^ Adverse effects, which include learning disabilities, developmental delays, cognitive impairments, and sensory deficiencies, could result from nutritional shortages that manifest during critical phases of brain development.^26^ Asymptomatic injuries that develop during the early stages of brain development appear to have long-lasting consequences for health in adulthood.^27^ Moreover, it is thought that the observed cognitive alterations interact with the divergence that occurs in the developmental pathway of some brain regions during childhood.^28^ To better prevent late-life morbidities, Lazar and colleagues proposed that intervention approaches that focus on modifiable risk and protective factors may be put into practice as early as childhood or young adulthood.^29,30^

Our findings highlight a significant association between the perception of different brain diseases/disorders and variables of age, gender and educational level. Despite the negative effect of migraine on brain performance and quality of life, study respondents (aged 41 years and older) were less likely to perceive that migraine is associated with brain health. Respondents’ perception may arise from the thought that migraine is just a minor inconvenience, and this could result in delayed treatment seeking. Multiple investigations have shown how vascular variables affect cognitive health in later life, particularly diabetes mellitus^31^ and hypertension.^32^ Iadecola et al.^33^ suggest that treating hypertension and other vascular risk factors early in life may both reduce the need for and increase the safety of antihypertensive medication. However, having an uninformed perception of the impact of hypertension on brain wellness may result in poor management of blood pressure, thereby increasing the risk of adverse outcomes for brain health. There is compelling evidence that brain disorders such as dementia, including Alzheimer’s, mixed-type dementia, and multi-infarct dementia, are more prevalent among individuals with type 2 diabetes.^34^ For a healthy brain, it is therefore imperative, to prioritise blood sugar, blood pressure, and cholesterol management, social and physical exercise, and moderation in alcohol consumption, weight control, and having enough sleep.^35^

Overall, numerous barriers could hinder effective communication about brain health in Nigeria. These may include language barriers and varying levels of health literacy among the population. To promote awareness and correct the wrong perceptions about brain health, a collaborative effort from governmental and non-governmental institutions is required. Nigeria can make substantial advances in enhancing public awareness of brain health by addressing educational, cultural, and socio-economic barriers and improving access to healthcare. Additionally, maximising the use of digital technologies in advancing public awareness of brain health could provide opportunities to promote the knowledge of potentially remediable risk factors of brain health.

This study presents some limitations that could be linked to the selected methodology. An online survey approach used for data collection may restrict the involvement of rural, non-literate and impoverished residents, who might have limited or no access to technology and the Internet. This also might have a skewed effect on our data, as most respondents were highly educated and from the Yoruba tribe. Additionally, response bias could be unavoidable as respondents may tend not to provide honest answers. Sampling issues because of the online research method applied limit the generalisability of this study. Future studies should focus on the inclusion of a more robust methodology aimed at achieving more reliable data. Despite these limitations, this study retains its validity as it directly captured first-hand information on perceptions of brain health in Nigeria. Its relevance lies in serving as a preliminary baseline for a more robust research.

Our study highlighted knowledge gaps on issues related to brain health. This suggests the need for formulation and implementation of health policies that prioritises brain health awareness from the community level to the national level. This could inform the design of interventional strategies, including educational campaigns that are tailored to different demographics such as age, gender and educational level groups. It is imperative that a multisectoral pathway involving the clinicians, public health professionals and community stakeholders engage in community sensitisation and awareness campaigns on risk factors, early signs and symptoms, preventive measures and management of brain diseases and disorders for early detection and better treatment. In addition, to promote a healthy brain throughout the life span, the inclusion of brain health-related knowledge in primary and secondary education curricula is suggested to keep the younger population well-informed on risk and protective factors for brain health. Allocation of funds for brain health research should be prioritised to facilitate the gathering of data on the nation’s current state of awareness and knowledge. Urgent implementation of these interventions is needed to change uninformed brain health-related perceptions of individuals and communities, improve knowledge, and ultimately improve brain health across the nation.

## Conclusion

The study’s findings revealed a poor understanding among the study population regarding key contributors to brain disorders, such as substance use, hypertension and diabetes. This highlights the urgent need for action by the government, policymakers and relevant stakeholders. Given the critical impact of brain health on national development, prioritising health promotion programmes aimed at changing uninformed perceptions and behaviours towards fostering brain health is paramount. Future research should explore both positive and negative perceptions of specific brain diseases/disorders which may pinpoint attitudes towards discrimination and stigmatisation. This study additionally recommends extending brain health perception studies to other countries in Africa to identify gaps peculiar to each regional setting.

## References

[1] World Health Organization. Optimizing brain health across the life course: WHO position paper [homepage on the Internet]. 2022 [cited 2024 May 20]. Available from: https://www.who.int/health-topics/brain-health#tab=tab_1

[2] Steinmetz JD, Seeher KM, Schiess N, et al. Global, regional, and national burden of disorders affecting the nervous system, 1990–2021: A systematic analysis for the global burden of disease study 2021. Lancet Neurol. 20204;23(4):344–381. 10.1016/S1474-4422(24)00038-3 PMC10949203 38493795

[3] Institute for Health Metrics and Evaluation. Brain health atlas: Understanding brain health around the world [homepage on the Internet]. 2024 [cited 2024 May 20]. Available from: brainhealthatlas.org

[4] Feigin VL, Vos T, Nichols E, et al. The global burden of neurological disorders: Translating evidence into policy. Lancet Neurol. 2020;19(3):255–265. 10.1016/S1474-4422(19)30411-9 31813850 PMC9945815

[5] Gandy M, Karin E, Fogliati VJ, et al. Emotional and cognitive difficulties, help-seeking, and barriers to treatment in neurological disorders. Rehabil Psychol. 2018;63(4):563. 10.1037/rep0000241 30247055

[6] George-Carey R, Adeloye D, Chan KY, et al. An estimate of the prevalence of dementia in Africa: A systematic analysis. J Glob Health. 2012;2(2):020401. 10.7189/jogh.02.020401 23289076 PMC3529309

[7] El Tallawy HN, Farghaly WM, Metwaly NA, et al. Door-to-door survey of major neurological disorders in Al Kharga District, New Valley, Egypt: Methodological aspects. Neuroepidemiology. 2010;35(3):185–190. 10.1159/000314345 20664292

[8] Ochayi B, Thacher T. Risk factors for dementia in central Nigeria. Aging Ment Health. 2006;10(6):616–620. 10.1080/13607860600736182 17050090

[9] Citak TG, Citak BN, Cerit B. The relationship between international students’ health perceptions and their healthy lifestyle behaviors. J Relig Health. 2021;60(6):4331–4344. 10.1007/s10943-021-01336-0 34245435 PMC8272445

[10] Cations M, Radisic G, Crotty M, Laver KE. What does the general public understand about prevention and treatment of dementia? A systematic review of population-based surveys. PLoS One. 2018;13(4):e0196085. 10.1371/journal.pone.0196085 29672559 PMC5908164

[11] Budin-Ljøsne I, Mowinckel AM, Friedman BB, et al. Public perceptions of brain health: An international, online cross-sectional survey. BMJ Open. 2022;12(4):e057999. 10.1136/bmjopen-2021-057999 PMC9016409 35437254

[12] Balogun WG, Cobham AE, Amin A. Neuroscience in Nigeria: The past, the present and the future. Metab Brain Dis. 2018;33:359–368. 10.1007/s11011-017-0119-9 28993966

[13] Adeloye D, Auta A, Ezejimofor M, et al. Prevalence of dementia in Nigeria: A systematic review of the evidence. J Glob Health Rep. 2019;3:e2019014. 10.29392/joghr.3.e2019014 31528708 PMC6746335

[14] Otubogun FM, Akinyemi R, Ogunniyi S. Burden of adult neurological diseases in Odeda Area, Southwest Nigeria. BMJ Neurol Open. 2020;2(2):e000062. 10.1136/bmjno-2020-000062 PMC7903184 33681795

[15] Alebachew W, Semahegn A, Ali T, Mekonnen H. Prevalence, associated factors and consequences of substance use among health and medical science students of Haramaya University, eastern Ethiopia, 2018: A cross-sectional study. BMC Psychiatry. 2019;19(1):1–9. 10.1186/s12888-019-2340-z 31694591 PMC6836499

[16] Hosking DE, Sargent-Cox KA, Anstey KJ. An Australian survey of cognitive health beliefs, intentions, and behaviours through the adult life course. Prev Med Rep. 2015;2:498–504. 10.1016/j.pmedr.2015.06.008 26844109 PMC4721299

[17] McLellan AT. Substance misuse and substance use disorders: Why do they matter in healthcare? Trans Am Clin Climatol Assoc. 2017;128:112. 28790493 PMC5525418

[18] Squeglia LM, Gray KM. Alcohol and drug use and the developing brain. Curr Psychiatry Rep. 2016;18:1–10. 10.1007/s11920-016-0689-y 26984684 PMC4883014

[19] Sella E, Miola L, Toffalini E, Borella E. The relationship between sleep quality and quality of life in aging: A systematic review and meta-analysis. Health Psychol Rev. 2023;17(1):169–191. 10.1080/17437199.2021.1974309 34459704

[20] Wong ML, Lau EYY, Wan JHY, Cheung SF, Hui CH, Mok DSY. The interplay between sleep and mood in predicting academic functioning, physical health and psychological health: A longitudinal study. J Psychosom Res. 2013;74(4), 271–277. 10.1016/j.jpsychores.2012.08.014 23497826

[21] Watson NF, Martin JL, Wise MS, et al. Delaying middle school and high school start times promotes student health and performance: An American Academy of Sleep Medicine position statement. J Clin Sleep Med. 2017;13(4):623–625. 10.5664/jcsm.6558 28416043 PMC5359340

[22] Egbulonu KG, Eleonu IS. Gender inequality and economic growth in Nigeria (1990–2016). Int J Gend Women Stud. 2018;6(1):159–167. 10.15640/ijgws.v6n1a14

[23] Wilcox S, Sharkey JR, Mathews AE, et al. Perceptions and beliefs about the role of physical activity and nutrition on brain health in older adults. Gerontologist. 2009;49(S1):S61–S71. 10.1093/geront/gnp078 19525218

[24] Delgado CE. Undergraduate student awareness of issues related to preconception health and pregnancy. Matern Child Health J. 2008;12:774–782. 10.1007/s10995-007-0300-6 17975718

[25] Ishak SH, Yaacob LH, Ishak A. Knowledge of pre-pregnancy care among men attending the outpatient clinics of Hospital Universiti Sains Malaysia. Malays J Med Sci. 2021;28(2):119. 10.21315/mjms2021.28.2.11 33958966 PMC8075591

[26] Vohr BR, Poggi DE, Wanke CA, Krebs NF. Neurodevelopment: The impact of nutrition and inflammation during preconception and pregnancy in low-resource settings. Pediatrics. 2017;139(suppl_1):S38–S49. 10.1542/peds.2016-2828F 28562247

[27] Scheinost D, Sinha R, Cross SN, et al. Does prenatal stress alter the developing connectome? Pediatr Res. 2017;81(1):214–226. 10.1038/pr.2016.197 27673421 PMC5313513

[28] Moura L, Crossley N, Zugman A, et al. Coordinated brain development: Exploring the synchrony between changes in grey and white matter during childhood maturation. Brain Imaging Behav. 2017;11:808–817. 10.1007/s11682-016-9555-0 27169540

[29] Lazar RM, Howard VJ, Kernan WN, et al. A primary care agenda for brain health: A scientific statement from the American Heart Association. Stroke. 2021;52(6):e295–e308. 10.1161/STR.0000000000000367 33719523 PMC8995075

[30] Vos SJ, Van BMP, Schiepers OJ, et al. Modifiable risk factors for prevention of dementia in midlife, late life and the oldest-old: Validation of the LIBRA index. J Alzheimer’s Dis. 2017;58(2):537–547. 10.3233/JAD-161208 28453475

[31] Yaffe K, Weston AL, Blackwell T, Krueger KA. The metabolic syndrome and development of cognitive impairment among older women. Arch Neurol. 2009;66(3):324–328. 10.1001/archneurol.2008.566 19273750 PMC2685462

[32] Scullin MK, Le DT, Shelton JT. Healthy heart, healthy brain: Hypertension affects cognitive functioning in older age. Transl Iss Psychol Sci. 2017;3(4):328. 10.1037/tps0000131

[33] Iadecola C, Yaffe K, Biller J, et al. Impact of hypertension on cognitive function: A scientific statement from the American Heart Association. Hypertension. 2016;68(6):e67–e94. 10.1161/HYP.0000000000000053 27977393 PMC5361411

[34] Kravitz E, Schmeidler J, Beeri MS. Type 2 diabetes and cognitive compromise: Potential roles of diabetes-related therapies. Endocrinol Metabol Clin. 2013;42(3):489–501. 10.1016/j.ecl.2013.05.009 PMC3767929 24011882

[35] Kleindorfer DO, Towfighi A, Chaturvedi S, et al. 2021 guideline for the prevention of stroke in patients with stroke and transient ischemic attack: A guideline from the American Heart Association/American Stroke Association. Stroke. 2021;52(7):e364–e467. 10.1161/STR.0000000000000375 34024117

